# Joint modeling of linkage and association using affected sib-pair data

**DOI:** 10.1186/1753-6561-1-s1-s38

**Published:** 2007-12-18

**Authors:** Ming-Huei Chen, Jing Cui, Chao-Yu Guo, L Adrienne Cupples, Paul Van Eerdewegh, Josée Dupuis, Qiong Yang

**Affiliations:** 1 Department of Mathematics and Statistics, Boston University, 111 Cummington Street, Boston, Massachusetts 02115, USA; 2 Department of Medicine, Brigham and Women's Hospital, Harvard Medical School, 221 Longwood Avenue 341G, Boston, Massachusetts 02115, USA; 3 Department of Biostatistics, Boston University School of Public Health, 715 Albany Street, Boston, Massachusetts 02118, USA; 4 Genizon BioSciences Inc., 880 McCaffrey, Montreal, Quebec H4T 2C7, Canada

## Abstract

There has been a growing interest in developing strategies for identifying single-nucleotide polymorphisms (SNPs) that explain a linkage signal by joint modeling of linkage and association. We compare several existing methods and propose a new method called the homozygote sharing transmission-disequilibrium test (HSTDT) to detect linkage and association or to identify SNPs explaining the linkage signal on chromosome 6 for rheumatoid arthritis using 100 replicates of the Genetic Analysis Workshop (GAW) 15 simulated affected sib-pair data. Existing methods considered included the family-based tests of association implemented in FBAT, a transmission-disequilibrium test, a conditional logistic regression approach, a likelihood-based approach implemented in LAMP, and the homozygote sharing test (HST). We compared the type I error rates and power for tests classified into three categories according to their null hypotheses: 1) no association in the presence of linkage (i.e., a SNP explains none of the linkage evidence), 2) no linkage adjusting for the association (i.e., a SNP explains all linkage evidence), and 3) no linkage and no association. For testing association in the presence of linkage, we found similar power among all tests except for the homozygote sharing test that had lower power. When testing linkage adjusting for association, similar power was observed between LAMP and HST, but lower power for the conditional logistic regression method. When testing linkage or association, the conditional logistic regression method was more powerful than FBAT.

## Background

The availability of high throughput single-nucleotide polymorphism (SNP) genotyping technologies at more affordable costs has generated increasing enthusiasm for genome-wide association study (GWAS) for a wide range of disorders [1]. How to best analyze dense SNP data is of great interest to the scientific community. Family-based association tests model both linkage and association and thus can better localize the disease locus than linkage analyses alone and avoid spurious association results due to population admixture. Recently, there has been a growing interest in developing methods for identifying SNPs that account for all the observed linkage evidence [2], a goal that can be achieved by joint modeling of linkage and association.

There are three major types of family-based association tests categorized by their null hypotheses: 1) H_0_: no association, in the presence of linkage or the tested SNP is in linkage equilibrium (LE) with all disease loci (denoted T_1_); 2) H_0_: no linkage adjusting for the association, or the tested SNP is in complete linkage disequilibrium (LD) (*r*^2 ^= 1) with all disease loci (denoted T_2_); 3) H_0_: no linkage and no association (denoted T_3_). It is vital to understand the differences among these hypotheses and the relative efficiencies of valid tests for each hypothesis. In what follows, we classify the tests we considered into the three categories and compare the power and type I error among them.

## Methods

We used all 100 replicates of Genetic Analysis Workshop 15 (GAW15) simulated nuclear families, each containing both parents and two affected children (Problem 3). Rheumatoid arthritis is the phenotype in all of our analyses. An initial nonparametric multipoint genome-wide linkage scan [3] using SNP markers was performed with Merlin [4]. A linkage peak with mean logarithm of odds (LOD) score of 79 (from 100 replicates) at about 50 cM on chromosome 6 was identified. Because this linkage region is broad, we selected 2102 dense SNPs between 40 and 60 cM under the linkage peak for assessing power of all methods. In addition, we selected 947 dense SNPs between 130 and 140 cM on chromosome 6 for evaluating type I error rates of the null hypothesis T_1_. Although the LOD scores range from 3 to 6, these 947 SNPs are far away from the disease loci (DR, C and D locus) and should be in LE with the disease loci. For T_3_, type I error rates were evaluated using all 16 chromosomes that do not harbor any disease loci. Available data from controls were used to determine LD, as measured by *r*^2 ^between each of 2102 dense SNPs and the disease loci. For the tri-allelic DR locus, a generalized *R*^2 ^was calculated for each SNP and tested using the LOGISTIC procedure in SAS version 8 [5]. For the C and D loci, *r*^2 ^was estimated using Haploview [6] and tested using the method proposed by Sabatti and Risch [7]. For each SNP, the mean *r*^2 ^from the 100 replicates was used to estimate more accurately LD with the disease loci. We briefly describe and categorize each method by its hypotheses. The analyses were performed with knowledge of the "answers".

### Family-based association test (FBAT)

Rabinowitz and Laird [8] proposed the family-based association test (FBAT) that is applicable to multiple siblings, quantitative traits, and incomplete parental genotypes. FBAT is a valid test of T_3_. Lake et al. [9] extended FBAT to a valid test of T_1 _by incorporating an empirical variance estimate (FBAT-e).

### Conditional logistic regression method

Millstein et al. [10] recently proposed a pseudo-control approach for joint modeling of linkage and association. Let g_1_, g_2_, g_m_, g_f _denote the genotypes at a studied locus for two affected offspring, mother, and father, respectively. D_1 _and D_2 _are the disease states for the two sibs. Conditional on parental genotypes and their disease states, the likelihood for the children is

*P*(*g*_1_, *g*_2_|*g*_*m*_, *g*_*f*_, *D*_1_, *D*_2_) = *P*(*g*_1_|*g*_*m*_, *g*_*f*_, *D*_1_) × *P*(*g*_2_|*g*_1_, *g*_*m*_, *g*_*f*_, *D*_1_, *D*_2_),

which can be modelled as

$$\frac{e^{\beta g_{1}}}{\sum_{g^{*}}e^{\beta g^{*}}}\times \frac{e^{\beta g_{2}+\gamma e_{12}}}{\sum_{g^{*}}e^{\beta g^{*}+\gamma e_{1*}}},$$

where g* represents the four possible offspring genotypes; *e*_12 _= E[ibd_12_|g_1_, g_2_, g_m_, g_f_], the expected identical-by-decent (IBD) sharing between g_1 _and g_2 _given the observed marker genotypes and *e*_1* _is the expected IBD sharing between g_1 _and $g_{2}^{*}$. A test of *β *= 0 is a test of T_1 _(denoted Millstein-b), a test of *γ *= 0 is a test of T_2 _(denoted Millstein-c), and the two degree of freedom likelihood ratio test (LRT) of *β *= 0 and *γ *= 0 is a test of T_3 _(denoted Millstein-a).

### Likelihood-based approach – LAMP

Li et al. [2] proposed a method to identify SNPs in LD with the disease locus through estimation of the degree of LD between the tested SNP and the putative disease locus. The method is implemented in the software called LAMP. They use a likelihood function that 1) assumes a single di-allelic disease locus, 2) assumes no recombination between the tested SNP and the disease locus, 3) uses disease-SNP haplotype frequencies and disease penetrances as parameters, and 4) can incorporate information from flanking markers in LE with the tested SNP. Two LRTs are proposed. The first (denoted as LAMP-LE) assesses whether the tested SNP is in LE with the disease locus, while the second (denoted as LAMP-LD) assesses whether the tested SNP is in complete LD with the disease locus. Therefore, LAMP-LE is a test of T_1 _and LAMP-LD is a test of T_2_. The statistical significance of these two tests is assessed empirically by comparing the observed statistic with simulated null distributions. We exclude flanking short tandem repeat (STR) markers in LD [7] with tested SNPs and use the remaining STRs in our application of LAMP.

### Homozygote Sharing Test (HST)

The HST statistic [11,12] is constructed using a likelihood function conditional on parental genotypes. It compares the observed IBD sharing from homozygous and heterozygous parents to determine if a SNP explains partially the evidence for linkage. HST capitalizes on the fact that parents who are homozygous at all disease loci in a linkage region should not transmit any alleles preferentially to the affected siblings, and hence no excess IBD sharing should be observed from homozygous parents. Additionally, the IBD sharing from homozygous and heterozygous parents should be equal for SNPs in LE with all disease loci. For the intermediate case in which the tested SNP is in partial LD with disease loci, some increased sharing may be observed from homozygous parents in a linkage region. The HST statistic to identify SNPs explaining some of the linkage evidence is derived from the likelihood ratio of the following hypotheses H_0_:1/2 <*α*_*homo *_= *α*_*het *_vs. H_1_: 1/2 ≤ *α*_*homo *_<*α*_*het*_, where *α*_*homo *_and *α*_*het *_are the probabilities that an affected sib-pair shares one allele IBD with respect to homozygous and heterozygous parents, respectively. The HST is defined as

$$\begin{array}{lll} HST & = & 2[N_{1}^{homo}log\left(\frac{N_{1}^{homo}}{N^{homo}}\right)+N_{0}^{homo}log\left(\frac{N_{0}^{homo}}{N^{homo}}\right)+N_{1}^{het}log\left(\frac{N_{1}^{het}}{N^{het}}\right)+N_{0}^{het}log\left(\frac{N_{0}^{het}}{N^{het}}\right)\\ & - & \left(N_{1}^{homo}+N_{1}^{het}\right)log\left(\frac{N_{1}^{homo}+N_{1}^{het}}{N^{homo}+N^{het}}\right)-\left(N_{0}^{homo}+N_{0}^{het}\right)log\left(\frac{N_{0}^{homo}+N_{0}^{het}}{N^{homo}+N^{het}}\right)], \end{array}$$

where $N_{j}^{homo}$ and $N_{j}^{het}$ denote the number of sib pairs sharing "*j*" allele IBD from homozygous and heterozygous parents respectively (*j *= 0,1). This HST statistic (denoted HST-LE) is a test of T_1_. Once subsets of SNPs explaining some of the linkage evidence have been identified, one can then test H_0_: 1/2 = *α*_*homo *_<*α*_*het *_vs. H_1_: 1/2 <*α*_*homo *_<*α*_*het *_with the following HST statistic:

$$2\left[N_{1}^{homo}log\left(\frac{N_{1}^{homo}}{N^{homo}}\right)+N_{0}^{homo}log\left(\frac{N_{0}^{homo}}{N^{homo}}\right)-N^{homo}log\left(\frac{1}{2}\right)\right].$$

Rejection of the null hypothesis indicates that the tested SNP does not explain fully the linkage evidence. This HST statistic (denoted HST-LD) is a test of T_2_. Both HST-LE and HST-LD are LRTs under independent parental transmissions, equivalent to assuming a multiplicative model of transmission. Under the null hypothesis of LE between the tested SNP and disease loci, both HST statistics asymptotically follow a chi-square mixture distribution of $0.5\chi_{0}^{2}+0.5\chi_{1}^{2}$, assuming independent parental transmissions.

### HSTDT – Combination of HST-LE and transmission-disequilibrium test (TDT)

The original TDT, proposed by Spielman et al. [13], tests linkage and association between a marker and a disease locus using ascertained affected individuals and his/her parental marker information. The TDT can also be used to test association in a linked region (Spielman and Ewens [14]) using data that consist of nuclear families with a single affected child. The TDT examines the allelic transmission to the affected child from his/her heterozygous parents. For families with multiple affected siblings, the transmissions are correlated among siblings if there is linkage, and TDT is no longer a valid test of association in the presence of linkage. To solve this problem, Martin et al. [15] focused on the set of transmissions from a heterozygous parent shared by all his/her affected children. For affected sib-pair data and a marker with two alleles *M*_1 _and *M*_2_, they showed that for a marker in LE with the disease loci, the probability that both affected siblings receive *M*_1 _(denoted as $\alpha_{het}^{M_{1}}$) and the probability that both affected siblings receive *M*_2 _(denoted as $\alpha_{het}^{M_{2}}$) from their heterozygous parent are equal. Thus, there should be no over-transmission of *M*_1 _or *M*_2 _to affected offspring. In what follows we use TDT to refer Martin et al.'s strategy, which is a test of T_1 _[15]. Note that the TDT does not use information from homozygous parents, while HST-LE compares the observed allele sharing from homozygous and heterozygous parents without considering which allele is over-transmitted from heterozygous parents. To fully use all available information to identify whether a SNP explains some of the linkage evidence, we propose HSTDT, which combines HST-LE and TDT by decomposing the allele sharing from heterozygous parents (*α*_*het*_) into two allele-specific IBD sharing probabilities ($\alpha_{het}^{M_{1}}$ and $\alpha_{het}^{M_{2}}$), to test H_0_: $1/2<\alpha_{homo}=\alpha_{het}=2\alpha_{het}^{M_{1}}=2\alpha_{het}^{M_{2}}$ vs. H_1_: $1/2\leq \alpha_{homo}<\alpha_{het},\alpha_{het}^{M_{1}}\neq \alpha_{het}^{M_{2}}$. The HSTDT statistic is defined as

$$\begin{array}{lll} \text{HSTDT} & = & 2[N_{1}^{homo}\log\left(\frac{N_{1}^{homo}}{N^{homo}}\right)+N_{0}^{homo}\log\left(\frac{N_{0}^{homo}}{N^{homo}}\right)+N_{1^{M_{1}}}^{het}\log\left(\frac{N_{1^{M_{1}}}^{het}}{N^{het}}\right)+N_{1^{M_{2}}}^{het}\log\left(\frac{N_{1^{M_{2}}}^{het}}{N^{het}}\right)\\ & + & N_{0}^{het}\log\left(\frac{N_{0}^{het}}{N^{het}}\right)+N_{1}^{het}\log2-\left(N_{1}^{homo}+N_{1}^{het}\right)\log\left(\frac{N_{1}^{homo}+N_{1}^{het}}{N^{homo}+N^{het}}\right)\\ & - & ⤢⤢⤢\left(N_{0}^{homo}+N_{0}^{het}\right)\log\left(\frac{N_{0}^{homo}+N_{0}^{het}}{N^{homo}+N^{het}}\right)]. \end{array}$$

Similar to HST-LE, HSTDT is a LRT under the assumption of independent parental transmission. Under the null hypothesis of LE between the tested SNP and disease loci (T_1_), the HSTDT asymptotically follows a chi-square mixture distribution of $0.5\chi_{1}^{2}+0.5\chi_{2}^{2}$, assuming independent parental transmissions.

The major distinction between homozygote sharing tests (HST and HSTDT) and other tests of T_1 _or T_2 _is that the former can be used to test if a SNP explains the linkage peak (by using IBD information at the linkage peak). When assuming no recombination between the tested SNP and the presumed disease locus at the linkage peak, testing association in the presence of linkage is equivalent to testing whether a SNP partially explains the linkage peak; while testing no linkage, adjusting for association is equivalent to testing whether the tested SNP fully explains the linkage peak. However, when the assumption is violated, for tests of T_1 _and T_2 _other than HST and HSTDT, one may not be able to claim that the tested SNP explains the peak linkage evidence but rather that it explains the linkage evidence at the location of the tested SNP. When a linkage signal is identified in a linked region, the LOD score at the linkage peak should be of greatest interest and is the usual quantity reported. In this report, HST and HSTDT are applied to identify SNPs explaining the peak linkage evidence.

## Results

SNPs were classified into five groups according to their LD with the disease loci. In Table 1, the first group (labeled *r*^2 ^= 0) included 947 dense SNPs between 130 and 140 cM on chromosome 6 that were used to assess type I error rates for T_1_. In Table 2, the first group (labeled *r*^2 ^= 0, *θ *= 0.5) included 6597 SNPs from all 16 chromosomes that did not harbor any disease loci and were used to assess type I error rates for T_3_. The remaining SNPs were grouped by the maximum of the three mean *r*^2 ^values with the three disease loci (C, D, and DR). For example, the group 0.1 <*r*^2 ^<= 0.3 included SNPs with mean *r*^2 ^between 0.1 and 0.3 with at least one of the disease loci. Power and type I error rates were assessed at significance levels of 5%, 1%, and 0.1%.

**Table 1:** Type I error rates and power (in percentage) for testing association in the presence of linkage (T_1_)

		LD group (number of SNPs)				
		
	Significance level (%)	*r*^2 ^= 0 (*n *= 947)	0 <*r*^2 ^<= 0.01 (*n *= 1751)	0.01 <*r*^2 ^<= 0.1 (*n *= 304)	0.1 <*r*^2 ^<= 0.3 (*n *= 29)	0.3 <*r*^2 ^<= 0.92 (*n *= 18)
FBAT-e	5	4.9	29.1	90.4	100	100
	1	1	18.4	85.3	100	100
	0.1	0.1	10.4	77.2	99.9	100
						
Millstein-b	5	4.9	28.6	90.1	100	100
	1	1	18	84.9	100	100
	0.1	0.1	10.1	76.6	99.9	100
						
LAMP-LE	5	4.3	22.5	87.8	100	100
	1	0.9	14.3	81.8	100	100
	0.1	0.1	8.2	73.4	99.8	100
						
TDT	5	5.1	29.9	90.7	100	100
	1	1.1	19.1	85.9	100	100
	0.1	0.1	11	78.1	99.9	100
						
HST-LE	5	5.2	5.9	21.3	49.9	86.9
	1	1.1	1.4	10.3	41.8	79.5
	0.1	0.1	0.2	4.1	34.1	74.9
						
HSTDT	5	7.3	29.2	90.2	100	100
	1	1.9	18.6	85.3	100	100
	0.1	0.3	11	77.9	99.8	100

**Table 2:** Type I error rates and power (in percentage)for testing linkage or association (T_3_)

		LD group (number of SNPs)				
		
	Significance level (%)	*r*^2 ^= 0, *θ *= 0.5 (*n *= 6597)	0 <*r*^2 ^<= 0.01 (*n *= 1751)	0.01 <*r*^2 ^<= 0.1 (*n *= 304)	0.1 <*r*^2 ^<= 0.3 (*n *= 29)	0.3 <*r*^2 ^<= 0.92 (*n *= 18)
FBAT	5	5	34.8	92.1	100	100
	1	1	23.7	88.4	100	100
	0.1	0.1	15	82.6	100	100
						
Millstein-a	5	5	99.2	100	100	100
	1	1	98.2	100	100	100
	0.1	0.1	96.3	99.9	100	100

### Testing association in the presence of linkage (T_1_)

Table 1 presents results for tests of T_1_. All methods have appropriate type I error rates (*r*^2 ^= 0), except that HSTDT has slightly inflated type I error rates. TDT is the most powerful among these six methods, followed closely by HSTDT, FBAT-e, Millstein-b and then LAMP-LE. HST-LE appears to be less powerful.

### Testing linkage adjusted for association (T_2_)

Table 3 presents results for tests of T_2_. These tests are used to examine if a SNP explains all the observed linkage evidence. LAMP-LD and HST-LD test whether a SNP is in complete LD with the disease loci. Equivalently, Millstein-c tests if there is residual linkage when conditioning on the genotype covariate of the tested SNP and is essentially a binary-trait version of the test proposed by Almasy and Blangero [16]. Because none of the SNPs is in complete LD with all disease loci, type I error rates cannot be evaluated. HST-LD and LAMP-LD have similar power and are more powerful than Millstein-c.

**Table 3:** Power (in percentage) for testing linkage adjusted for association (T_2_)

		LD group (number of SNPs)			
		
	Significance level (%)	0 <*r*^2 ^<= 0.01 (*n *= 1751)	0.01 <*r*^2 ^<= 0.1 (*n *= 304)	0.1 <*r*^2 ^<= 0.3 (*n *= 29)	0.3 <*r*^2 ^<= 0.92 (*n *= 18)
Millstein-c	5	97.3	97.9	97.1	75.6
	1	95.4	96.5	93.1	62.7
	0.1	93	94.8	85.9	50.8
					
LAMP-LD	5	100	100	99.9	99.3
	1	100	100	99.8	98.8
	0.1	100	100	99.8	97.2
					
HST-LD	5	100	100	100	96.6
	1	100	100	100	96.6
	0.1	100	100	100	93.2

### Testing linkage or association (T_3_)

Table 2 presents results for tests of T_3_. The type I error rates (*r*^2 ^= 0, *θ *= 0.5) of FBAT and Millstein-a are appropriate. FBAT is less powerful than Millstein-a for SNPs in low LD with the disease loci. Note that Millstein-a directly uses IBD between sib pairs in the model, while FBAT uses allelic transmission from parents to children, possibly explaining the difference in their ability to pick up the linkage signal.

## Conclusion

With a dense SNP map, it is natural to speculate whether all disease loci under a linkage peak have been identified or their contributions to the phenotypic variation are fully explained by a small subset of SNPs in association with these disease loci. Recently, there was much interest in testing whether a SNP can partially or fully account for all the observed linkage evidence.

We examined several methods for joint linkage and association analysis and identifying SNPs that explain the linkage evidence. For testing association in the presence of linkage and for testing linkage or association, all methods have appropriate type I error rates. For testing association in the presence of linkage, TDT is most powerful (but only slightly more powerful than HSTDT, FBAT-e, Millstein-b, and LAMP). For testing whether a SNP explains all the linkage evidence, HST and LAMP have similar power and are more powerful than Millstein-a; the difference in power may be explained by the difference in type I error, which we were unable to assess in the GAW15 data set because there was no single disease locus explaining all of the linkage evidence. For testing linkage or association, we found that Millstein-c was more powerful than FBAT. These conclusions may not extend to study designs other than nuclear families each with two affected children with parental genotypes available, a requirement for HST, HSTDT, and Millstein et al. [10] Furthermore, the excessively high LOD score observed in this study may explain the slightly inflated type I error rate observed for HSTDT.

In this study, there are three disease loci in the linked region and they do not contribute equally to the linkage signal, with the DR locus having a major effect on affection status. A different scenario may lead to different results for methods that use linkage peak information to identify SNPs, explaining the linkage evidence. However, for a complex disease, there may be multiple disease loci acting interactively, so methods that do not assume a single causal variant would be most helpful in identifying SNPs associated with disease loci. There is a great need for developing methods suitable for multiple disease loci.

## Competing interests

The author(s) declare that they have no competing interests.
